# Dynapaenic Abdominal Obesity Is Associated With a Poorer Quality of Life in Women Diagnosed With Breast Cancer Awaiting Surgery

**DOI:** 10.1155/jobe/4826660

**Published:** 2026-10-17

**Authors:** Taise Andrade da Anunciação, Anna Karla Carneiro Roriz, Maria Lúcia Varjão da Costa, Viviane Sahade Souza, Rosângela Passos de Jesus, Priscila Ribas Farias Costa, Allain A. Bueno, Lucivalda Pereira Magalhães de Oliveira

**Affiliations:** ^1^ Post-Graduate Programme in Food Nutrition and Health School of Nutrition at the Federal University of Bahia Salvador Brazil ufba.br; ^2^ Health Sciences Centre Federal University of Recôncavo da Bahia Santo Antônio de Jesus Brazil lhsc.on.ca; ^3^ Department of Nutrition Graduate Programme in Food Nutrition and Health School of Nutrition Federal University of Bahia Salvador Brazil ufba.br; ^4^ Nutritionist at Hospital Aristides Maltez Salvador Brazil; ^5^ College of Health Life and Environmental Sciences University of Worcester Worcester WR2 6AJ UK worcester.ac.uk

**Keywords:** abdominal obesity, body composition, breast cancer, dynapaenia, muscle strength, quality of life

## Abstract

**Background:**

Lower quality of life (QoL) in cancer can negatively influence survival and treatment course. We have investigated the co‐occurrence of dynapaenia and overweight or obesity in a sample population of women diagnosed with breast cancer admitted for cancer surgical treatment and how those chronic conditions influence QoL.

**Methods:**

Ninety‐seven overweight or obese women admitted to hospital for scheduled lumpectomy or mastectomy were recruited. Correlative analyses of clinical, epidemiological, anthropometric, nutritional, and handgrip strength assessments were carried out. Dynapaenic abdominal obesity was defined as the coexistence of BMI ≥ 30 kg/m^2^, waist circumference ≥ 88 cm, and dynapaenia.

**Results:**

The prevalence of dynapaenic abdominal obesity was associated with significantly lower QoL scores. Moreover, women with lower education, irregular physical activity, lower income and more severe obesity showed worsened QoL in the Functional Assessment of Cancer Therapy–Breast and–General domains and respective subdomains and in the Trial Outcome Index.

**Conclusions:**

Dynapaenic abdominal obesity was independently associated with poorer QoL in women with breast cancer awaiting surgery, even after adjustment for age, income, education and physical activity. Our findings raise the possibility that the coexistence of dynapaenic abdominal obesity with cancer may have an additive or synergistic detrimental effect on health outcomes. As QoL is associated with health outcomes in breast cancer, early clinical assessment of muscle strength, fat distribution, and nutritional status may help identify patients with greater vulnerability and support more comprehensive care strategies.

## 1. Introduction

Obesity is a well‐established risk factor for various types of cancer, including breast cancer [1]. Metabolic derangements caused by the overall burden of obesity on cancer patients, added to the undesired effects of antineoplastic therapies, often lead to the development of dynapaenia or sarcopenia [2]. Sarcopenia involves the progressive loss of both muscle mass and strength, while dynapaenia is defined as reduced muscle power without a necessarily corresponding loss of muscle mass [3].

Sarcopenia is known to increase mortality risk in older adults [4]. However, clinical studies have suggested that dynapaenia is more prevalent than sarcopenia and may precede nutritional risk [5, 6]. The co‐occurrence of dynapaenia and obesity in women affected by breast cancer is associated with worsened outcomes, including elevated risk of secondary malignancies, metastasis, delayed diagnosis, surgical complications, and poorer responses to treatment [3, 7].

In addition to metabolic derangements, obesity exacerbates musculoskeletal alterations, potentially due to elevated proinflammatory cytokines [8]. Increased adiposity has been shown to impair physical function, negatively impacting overall quality of life (QoL) [9]. Furthermore, obesity‐induced fat infiltration into muscle tissue significantly compromises muscle strength [10].

Experimental evidence suggests that muscle dysfunction in breast cancer patients may not be driven solely by changes in body weight or muscle mass but could also occur independently in antineoplastic therapies [11]. The same study showed that interventions targeting muscle mass and function may help alleviate fatigue and improve QoL [11].

Although diverse aetiologies explain the prognosis of dynapaenia and sarcopenia, our understanding of the interactions between dynapaenia and obesity in breast cancer patients remains limited [2]. Moreover, as low QoL in cancer can negatively influence survival and treatment course, expanding our knowledge of the associations between dynapaenia and obesity in breast cancer patients could not only contribute to enhancement of QoL and improved clinical outcomes for affected individuals but also guide more tailored preventative strategies for vulnerable populations. Our study investigated in women diagnosed with breast cancer admitted to hospital for scheduled lumpectomy or mastectomy the co‐occurrence of dynapaenia and abdominal obesity and its impact on QoL.

## 2. Methods

### 2.1. Study Design and Participants

This cross‐sectional study was approved by the Ethics and Research Committee of the Liga Bahiana Contra o Câncer—Aristides Maltez Hospital (opinion 2.950.264/2018).

All included participants had a confirmed diagnosis of breast cancer and had been admitted to a tertiary referral hospital in Salvador (Brazil) for a scheduled lumpectomy or mastectomy when they took part in the study. Eligible participants included women aged 18 years and older with a diagnosis of overweight or obesity. Non‐inclusion criteria included severe oedema, physical or cognitive limitations that could influence physical, anthropometric and nutritional assessments and interviews.

### 2.2. Data Collection

Data were collected by a team of trained nutritionists using a standardised protocol. Assessments included anthropometric measurements, muscle strength evaluation, nutritional assessment and QoL assessment. Covariates included age (adults 18–59 years vs*.* elderly ≥ 60 years), level of education (primary/secondary vs*.* high school/college/university), self‐declared ethnicity (Black, Asian and Minority Ethnic groups [BAME] vs*.* non‐BAME), monthly family income (defined as units of minimum wage, with each minimum wage unit in local currently equivalent to approximately US$183.2 at the time of the study), self‐reported physical activity habits (≥ 3 times a week vs*.* no exercise or 1‐2 times a week) and clinical variables (time of breast cancer diagnosis, phase of antineoplastic treatment, lymph node involvement, presence of metastasis, previous surgery).

Nutritional status was assessed using the Patient‐Generated Subjective Global Assessment (PG‐SGA) as previously standardised [12], under a licence for use (Pt‐Global Platform at https://www.pt-global.org). The PG‐SGA is recommended within the oncology community [13, 14] and for the purpose of our analysis, PG‐SGA scores of 9 or higher were considered indicative of nutritional risk.

### 2.3. Anthropometric Measurements

Weight was measured using a digital scale following guidance from the World Health Organization (WHO) [15]. Height for adults (18–59 years) was obtained using a portable stadiometer following WHO recommendations, while height for elderly women (≥ 60 years) was estimated by measuring knee height, as previously demonstrated [16]. Adult women were classified as overweight where BMI ≥ 25 kg/m^2^, and obese where BMI ≥ 30 kg/m^2^, according to WHO guidelines [15]. Elderly women were classified as overweight where BMI ≥ 28 kg/m^2^, and obese where BMI ≥ 30 kg/m^2^, according to classification by the Pan‐American Health Organization [17]. Waist circumference (WC) was measured twice at the midpoint between the lower rib and iliac crest [18] and defined as abdominally obese where values ≥ 88 cm, according to WHO [15].

### 2.4. Assessment of Dynapaenia

Dynapaenia was assessed by handgrip strength (HGS) using a Jamar dynamometer. Two measurements were taken on both hands, with the highest value used to determine maximum muscle contraction capacity. Our study protocol is based on the study of Caporrino et al. [19], which suggests cutoff points of the right or left arm for the Brazilian population. Readings from the right and left arms are relevant for our study to consider women who had a unilateral tumour and those who had undergone previous unilateral breast surgery on the opposite arm. Dynapaenia was defined as a reduction in HGS based on reference values for Brazilian adult women, adjusted for age [19] (Supporting table S1). Dynapaenia for elderly women was defined through guidance from the European Working Group on Sarcopenia in Older People (EWGSO) consensus (< 16 kg) [10].

### 2.5. Dynapaenic Abdominal Obesity

Dynapaenic abdominal obesity was defined as the coexistence of BMI ≥ 30 kg/m^2^, WC ≥ 88 cm, and dynapaenia [20].

### 2.6. QoL

QoL was assessed using the Functional Assessment of Cancer Therapy–Breast (FACT–B) Version 4, under a use licence. The FACT–B is a multidimensional questionnaire with 37 questions divided into five domains: physical well‐being, social/family well‐being, emotional well‐being, functional well‐being and a breast cancer subscale [21]. By regrouping the FACT–B subscales, the scores for Functional Assessment of Cancer Therapy–General (FACT–G) and Trial Outcome Index (TOI) were identified. The FACT–G consists of 27 questions grouped across four domains, similar to the FACT–B but without the breast cancer subscale. The TOI score is composed of 23 items extracted from the physical well‐being, functional well‐being and breast cancer subscales. Responses are scored on a 5‐point Likert scale, with a maximum possible FACT–B score of 148 points. Higher scores indicate better QoL.

### 2.7. Statistical Analyses

Descriptive statistics, including frequencies, means and standard deviations, were used to summarise results. The Shapiro–Wilk test was employed to assess data normality. Pearson’s chi‐square test and Fisher’s exact test evaluated associations between dynapaenic obesity and nondynapaenic obesity with other covariates. For normally distributed data, the Student’s *t*‐test was used to compare QoL subdomain mean scores (FACT–B) with the evaluated variables, namely self‐declared ethnicity, education, monthly family income, regular physical exercise, PG‐SGA, BMI, dynapaenic obesity and nondynapaenic obesity.

Ordinary least square (OLS) multiple linear regression models were used to test associations between dynapaenic obesity and QoL. The coefficient of determination (R^2^) was employed to measure the explanatory capacity of the model. The Akaike information criterion (AIC) was used to evaluate model fitness [22]. All statistical analyses were conducted using Stata/IC Version 16.0, with a significance level of 0.05 applied to all tests. Other covariates, such as treatment phase, time since diagnosis, lymph node involvement, presence of metastasis, previous treatments and climacteric phase, were evaluated as potential confounders.

## 3. Results

Ninety‐seven women provided consent and agreed to participate in the study. The average age was 52.2 ± 11.2 years. The BMI overall average was 31.6 ± 4.5 kg/m^2^, and the WC overall average was 97.8 ± 10.68 cm. Obesity was diagnosed in 56.7%, elevated WC was detected in 78.4%, dynapaenia was detected in 72.2%, and dynapaenic abdominal obesity was detected in 56.7% of participants (Table 1).

**TABLE 1:** Nutritional characteristics of women admitted to hospital for scheduled breast cancer surgery included in the investigation; Salvador, Bahia, Brazil.

Variables		
Age (years)	52.2 ± 11.2 (average ± SD)	

	***n***	**%**

*BMI (kg/* *m* ^2^ *)*		
Overweight	42	43.3
Obesity	55	56.7
*Waist circumference (cm)*		
Adequate	21	21.6
Increased	76	78.4
*Dynapaenia*		
Yes	70	72.2
No	27	27.8
*Dynapaenic obesity*		
Yes	55	56.7
No	42	43.3

*Note: N* = 97.

Abbreviation: BMI, body mass index.

Most participants had a spouse (52.6%), 86.6% self‐identified as Black, Brown, Indigenous or Asian (herein defined as Black, Asian and Minority Ethnic, BAME), 45.4% had completed high school or university, 76.1% reported low family income, 70.5% were nonsmokers, and 84.5% reported infrequent or no consumption of alcoholic beverages (Table 2). The diagnosis of breast cancer was given in the previous 6 months for 58.8% of participants, and 56.7% did not have any previous chemotherapy or radiotherapy treatment (Table 2).

**TABLE 2:** Sociodemographic, clinical and lifestyle characteristics according to dynapaenic obesity in women admitted to hospital for scheduled breast cancer surgery included in the investigation; Salvador, Bahia, Brazil.

Variables	*n* (%)	Dynapaenic obesity *n* (%)	No dynapaenic obesity *n* (%)	*p* value
*Age group*				
Adults	75 (77.3)	48 (87.3)	27 (64.3)	0.07^∗^
Elderly	22 (22.7)	7 (12.7)	15 (35.7)	

*Education*				
High school or university	44 (45.4)	24 (43.6)	20 (47.6)	0.43^∗^
Primary or secondary	53 (54.6)	31 (56.4)	22 (52.4)	

*Self-declared ethnicity*				
Non‐BAME	13 (13.4)	9 (16.4)	4 (9.5)	0.25^∗∗^
BAME	84 (86.6)	46 (83.6)	38 (90.5)	

Family income*^a^*				
≥ 2 SM	22 (23.9)	12 (23.5)	10 (24.4)	0.55^∗^
< 2 SM	70 (76.1)	39 (76.5)	31 (75.6)	

*Relationship status*				
No partnered	46 (47.4)	27 (49.1)	19 (45.2)	0.43^∗^
Partnered	51 (52.6)	28 (50.9)	23 (54.8)	

*Children*				
Yes	90 (92.8)	51 (92.7)	39 (92.9)	0.649^∗∗^
No	7 (7.2)	4 (7.3)	3 (7.1)	

*Regular physical exercise*				
Yes (≥ 3*x*/week)	23 (23.7)	10 (18.2)	13 (30.9)	0.11^∗^
No	74 (76.3)	45 (81.8)	29 (69.1)	

Smoking*^a^*				
Yes, or former smoker	28 (29.5)	15 (28.3)	13 (30.9)	0.47^∗^
No	67 (70.5)	38 (71.7)	29 (69.1)	

Alcohol frequency*^a^*				
Daily/weekly	14 (14.4)	6 (11.1)	8 (19.1)	0.21^∗^
No/rarely	82 (84.5)	48 (88.9)	34 (80.9)	

*Previous chemotherapy or radiotherapy treatment*				
Yes	42 (43.3)	23 (41.8)	19 (45.2)	0.44^∗^
No	55 (56.7)	32 (58.2)	23 (54.8)	

*Diagnosis time*				
< 6 months	57 (58.8)	33 (60.0)	24 (57.1)	0.92^∗^
≥ 6 months	40 (41.2)	22 (40.0)	18 (42.9)	

*PG-SGA*				
No nutritional risk	82 (84.5)	46 (83.6)	36 (85.7)	0.77^∗^
At nutritional risk	15 (15.5)	9 (16.4)	6 (14.3)	

*Note:* BAME: Black, Asian and Minority Ethnic groups; SM: minimum wage in Brazilian currency, equivalent to US$183.2 at the time of the study; PG‐SGA: Patient‐Generated Subjective Global Assessment. *N* = 97.

^∗^Pearson’s chi‐square test.

^∗∗^Fisher’s exact test.

^a^No data available (preferred not to say) as detailed: Family income: 5 women responded ‘prefer not to say’; Smoking: 2 women responded ‘prefer not to say’; alcohol frequency: 1 woman responded ‘prefer not to say’.

The reassessment of the aforementioned variables based on the diagnosis of dynapaenic abdominal obesity showed that a higher frequency of dynapaenic abdominal obesity was observed in women with lower, as compared to, higher educational levels (56.4% vs. 43.6%); in BAME participants, as compared to non‐BAME (83.6% vs. 16.4%); in those with lower income, as compared to higher (76.5% vs. 23.5%); in those who did not engage in regular physical exercise (81.8% vs. 18.2%); and in those at no nutritional risk (83.6% vs. 16.4%) (Table 2).

Supporting Table 1 presents the mean scores for the QoL subdomain questionnaires according to the characteristics of the population. BAME women had significantly higher scores in the physical subdomain compared to non‐BAME women (23.1 vs. 20.3, *p* = 0.04). Similarly, participants at no nutritional risk scored significantly higher in the physical subdomain than those at nutritional risk (23.4 vs. 18.9, *p* < 0.01).

In the social subdomain, women with secondary or higher education achieved significantly higher scores compared to those with only primary or secondary education (22.5 vs. 20.5, *p* = 0.01). Participants with a family income equal to or greater than two minimum wage units scored significantly higher in the social subdomain compared to those with lower income (22.9 vs. 21.2, *p* = 0.03) (Table 3).

**TABLE 3:** Mean of scores obtained from QoL questionnaires according to descriptive characteristics in women admitted to hospital for scheduled breast cancer surgery included in the investigation; Salvador, Bahia, Brazil.

	Dynapaenic obesity			No dynapaenic obesity		
	Scores			Scores		
	FACT–B Mean (SD)	FACT–G Mean (SD)	TOI Mean (SD)	FACT–B Mean (SD)	FACT–G Mean (SD)	TOI Mean (SD)
*Education*						
High school or university	108.8 (34.7)	85.8 (28.8)	63.6 (15.8)	115.1 (15.5)	90.7 (10.9)	69.4 (19.2)
Primary or secondary	100.0 (11.0)	76.4 (7.0)	62.9 (8.3)	111.9 (17.1)	84.2 (13.5)	71.3 (11.7)
*p* value	0.30	0.17	0.87	0.45	0.04	0.64

*Self-declared ethnicity*						
Non‐BAME	69.9 (42.5)	58.7 (31.5)	45.9 (19.9)	104.9 (15.5)	83.5 (15.1)	69.4 (13.7)
BAME	106.0 (22.3)	81.9 (19.1)	64.3 (10.9)	115.2 (16.0)	88.1 (12.1)	70.6 (16.05)
*p* value	0.04	0.11	0.03	0.05	0.27	0.81

*Family income*						
≥ 2 SM	98.3 (28.3)	79.2 (20.9)	60.0 (15.9)	115.3 (15.0)	91.6 (11.2)	71.2 (20.4)
< 2 SM	100.8 (12.6)	76.9 (8.1)	63.0 (10.7)	112.7 (16.6)	86.1 (12.5)	69.9 (14.1)
*p* value	0.72	0.66	0.55	0.61	0.13	0.80

*Regular physical exercise*						
Yes	101.3 (10.9)	77.7 (8.9)	62.8 (10.2)	115.7 (14.3)	89.9 (12.2)	76.3 (7.9)
No	104.3 (26.2)	81.0 (21.5)	63.3 (12.4)	112.4 (17.1)	86.2 (12.8)	68.0 (17.3)
*p* value	0.80	0.73	0.93	0.47	0.30	0.05

*PG-SGA*						
No risk	101.7 (12.3)	77.9 (7.9)	63.2 (11.0)	115.3 (15.3)	88.6 (12.3)	71.7 (15.7)
At risk	112.3 (50.2)	90.8 (42.3)	63.2 (16.2)	100.7 (18.0)	78.2 (12.4)	61.7 (11.6)
*p* value	0.31	0.13	0.99	0.01	0.03	0.09

*Note:* Student’s *t*‐test. Trial Outcome Index (TOI); Functional Assessment of General Cancer Therapy (FACT–G); Functional Assessment of Breast Cancer Therapy (FACT–B); BAME: Black, Asian and Minority Ethnic groups; SM: Minimum wage in Brazilian currency, equivalent to US$183.2 at the time of the study; PG‐SGA: Patient‐Generated Subjective Global Assessment. *N* = 97.

Abbreviation: BMI, body mass index.

In the functional subdomain, BAME women had significantly higher scores compared to non‐BAME women (20.7 vs. 16.0, *p* < 0.01). Obese women scored significantly lower in the emotional subdomain (18.2 vs. 20.6, *p* = 0.01), and in the breast cancer subscale (24.6 vs. 27.8, *p* < 0.01), compared to overweight women (Table 3). Overall, higher scores in each subdomain of the QoL questionnaire were associated with a greater likelihood of better QoL overall, as indicated by the total FACT–G score.

Table 3 presents the mean scores of the FACT–B, the FACT–G, and the TOI according to sociodemographic and clinical characteristics, stratified by the presence of dynapaenic abdominal obesity. Among women without dynapaenic abdominal obesity, participants with higher educational levels had significantly higher FACT–G scores, indicating better QoL, compared to those with lower educational levels (90.7 vs. 84.2, *p* = 0.04). In the same group, women classified as being at nutritional risk according to the PG‐SGA had significantly lower FACT–B and FACT–G scores compared to those without nutritional risk (100.7 vs. 115.3, *p* = 0.01; 78.2 vs. 88.6, *p* = 0.03, respectively). Regular physical exercise was associated with higher TOI scores among women without dynapaenic abdominal obesity (76.3 vs. 68.0, *p* = 0.05). Lower FACT–B and TOI scores were observed for dynapaenic abdominal obesity in non‐BAME participants compared to BAME participants (69.9 vs. 106.0, *p* = 0.04; 45.9 vs. 64.3, *p* = 0.03, respectively) (Table 3).

Women diagnosed with dynapaenic abdominal obesity exhibited significantly lower QoL scores across several subdomains compared to those without dynapaenic abdominal obesity (Figure 1). Specifically, in the physical well‐being (24.1 vs. 21.7, *p* = 0.01), emotional well‐being (20.5 vs. 18.3, *p* = 0.01), functional well‐being (21.6 vs. 18.8, *p* = 0.01) and the breast cancer subscale (27.9 vs. 24.6, *p* < 0.01) subdomains, as well as the TOI scores (73.7 vs. 65.2, *p* < 0.01), participants with dynapaenic obesity showed lower scores (Figure 1). Overall QoL scores, as measured by FACT–G and FACT–B, were markedly lower in women with dynapaenic abdominal obesity compared to those without (88.5 vs. 79.9, *p* < 0.01; 116.5 vs. 104.5, *p* < 0.01, respectively) (Figure 1).

**FIGURE 1:**
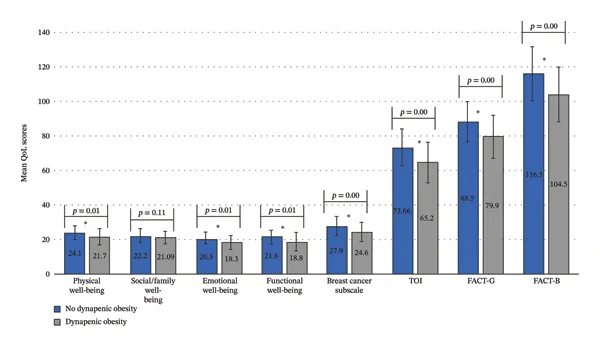
Quality of life subdomains of physical well‐being, social/family well‐being, emotional well‐being, functional well‐being, breast cancer subscale, TOI, FACT–G and FACT–B of women admitted to hospital for scheduled breast cancer surgery included in the investigation; Salvador, Bahia‐Brazil. TOI: Trial Outcome Index; FACT–G: Functional Assessment of Cancer Therapy–General; FACT–B: Functional Assessment of Cancer Therapy–Breast. ^∗^ *p* < 0.01.

Table 4 presents the association between dynapaenic abdominal obesity and total QoL scores measured by the FACT–B questionnaire. Women with dynapaenic abdominal obesity had mean total FACT–B scores that were 8.47 points lower than those without dynapaenic abdominal obesity (*β* = −8.47; 95% CI: −13.12 to −3.82; *p* < 0.001). After adjustment for age, income, educational level and physical activity, this association remained statistically significant, with women with dynapaenic abdominal obesity presenting total FACT–B scores that were 8.84 points lower than those without dynapaenic abdominal obesity (*β* = −8.84; 95% CI: −13.71 to −3.96; *p* = 0.001).

**TABLE 4:** Association between dynapaenic abdominal obesity and overall QoL, assessed by the total FACT–B score, in women admitted to hospital for scheduled breast cancer surgery included in the investigation; Salvador, Bahia, Brazil.

*β* coefficient (CI 95%)		
	Raw	Adjusted^†^
*Dynapaenic obesity*		
No	Reference	Reference
Yes	−8.47 (−13.12; −3.82); 0.000^∗^	−8.84 (−13.71; −3.96); 0.001^∗^
Adjusted R^2^ (%)	11.3	10.1
AIC	741.4	708.9

*Note:* Linear regression model, ^∗^ *p* < 0.01. *N* = 97.

Abbreviations: AIC, Akaike information criterion; CI, confidence interval.

^†^Adjusted for age, monthly family income, education and physical activity frequency.

## 4. Discussion

In the present study, we firstly investigated the co‐occurrence of dynapaenia and abdominal obesity in a sample population of overweight and obese women with a confirmed diagnosis of breast cancer, admitted to a tertiary referral hospital for scheduled lumpectomy or mastectomy. Subsequently, we examined associations between the coexistence of dynapaenic abdominal obesity with poorer QoL scores in the sample population recruited. Recruitment and data collection were performed at admission prior to surgery.

We found a high prevalence of dynapaenic abdominal obesity in our sample population, and a statistically significant association between the presence of dynapaenic abdominal obesity and lower QoL scores was detected. In summary, our results show that self‐declared BAME women, those with secondary and higher education, those who practise physical exercise more regularly, those earning a higher income, and those with a diagnosis of overweight as opposed to obesity, all presented higher scores in at least one of the QoL domains assessed (FACT–B total and subdomains Physical, Social/Family, Emotional, Functional, Breast Cancer Subscale; TOI; FACT–G) as compared to the respective comparison group. Our linear regression model raw results, and the results adjusted for age, monthly family income, education and physical activity frequency, all have shown that dynapaenic obese women presented lower QoL scores compared to those with nondynapaenic obesity.

For most participants, the hospital admission for cancer surgery in which this study was conducted was their first admission, which may suggest a lesser impact of cancer on QoL when compared to women admitted for their second or subsequent cancer interventions. It is known that women with symptomatic cancer have a worsened QoL as compared to asymptomatic counterparts [23], which corroborates our findings that dynapaenic obesity‐associated muscular changes were evident even in cases of recent breast cancer diagnosis, where participants had not yet undergone cancer treatment.

While previous studies with cancer sufferers [24] and with sufferers of other conditions [20] have identified detrimental effects of dynapaenic obesity on overall health, our study has identified novel deficits in specific QoL domains and subdomains which had not been explored before. Dynapaenia and obesity are very distinct conditions, but as previous studies have shown that both reduced muscle strength and obesity are independently associated with poorer physical function, increased fatigue, and lower QoL among women with breast cancer, as well as in populations with other chronic conditions [25–28], our findings support the hypothesis that their coexistence may have an additive or synergistic detrimental effect on health outcomes, although the isolated contribution of each component could not be determined in the present study.

Abdominal obesity has been associated with reduced muscle strength, an effect likely mediated by endocrine and proinflammatory mechanisms [5]. The elevated synthesis of proinflammatory cytokines observed in abdominal obesity is associated with accelerated decline of motor function, further elevating cardiovascular and metabolic risks. Moreover, abdominal obesity is associated with myosteatosis, resulting in anatomical and pathophysiological manifestations that ultimately lead to muscle weakness. Dynapaenic abdominal obesity is associated with cardiometabolic comorbidities, and more severe clinical outcomes have been observed in cancer patients diagnosed with dynapaenic abdominal obesity [2].

Skeletal muscle mass reduction and adipose mass increase have been simultaneously observed in breast cancer sufferers undergoing treatment and thereafter. Not only does it exacerbate inflammation, but also inadequate energy or protein intake, immobility, and physical disability can worsen breast cancer progression and reduce QoL [9, 29–31]. A clinical study [32] showed that women diagnosed with early‐stage breast cancer starting chemotherapy for Stage I to IIIa breast cancer were predominantly dynapaenic (54.5%), older, and overweight or obese. In the population investigated [32], the aforementioned characteristics were associated with worsened QoL, assessed by the Functional Assessment of Cancer Therapy–Anaemia and fatigue scale.

Sociodemographic, lifestyle, and anthropometric characteristics, as well as age, ethnicity, monthly family income, relationship status, sedentariness and obesity, are known players in chronic pain in women diagnosed with breast cancer, also negatively influencing QoL [33]. However, whilst questionnaire‐based techniques for sociodemographic and lifestyle screening can generate reliable information, the anthropometric assessment of dynapaenia and its association with obesity remains a contentious topic due to the lack of standardisation for the criteria used in different body composition assessment techniques [34]. Moreover, definitions and variability of cutoff points for the diagnosis of dynapaenic obesity are heterogenous, with many authors recommending WC as a reliable marker, given that BMI does not accurately reflect changes in body composition, particularly in a population that is already overweight [12].

By elucidating the association between dynapaenic abdominal obesity and poorer QoL in women diagnosed with breast cancer, our study contributes to a better understanding of the interaction between body composition, muscle strength and QoL. Fundamentally, the ability to perform daily activities and to engage socially in their environment are important components of QoL for women diagnosed with breast cancer.

Diagnosing and addressing dynapaenic abdominal obesity in the early stages of breast cancer may lead not only to the identification of women with greater vulnerability to poorer QoL but also to improved health outcomes and enhanced QoL for affected individuals by enabling early intervention and potentially contributing to a better prognosis. Therefore, incorporating assessments of muscle strength and body fat distribution into routine clinical practice is warranted to facilitate early intervention and optimise the management of breast cancer patients, in line with previous suggestions [6].

Our findings should be interpreted with caution, considering that the cross‐sectional design precludes causal inferences and determining the temporal direction of the observed associations. Moreover, given that multiple comparisons were performed across the FACT–B domains and summary scores, the domain‐specific results should be interpreted with caution and considered exploratory. In addition, the reference group was heterogeneous and included women with different combinations of overweight, obesity, abdominal adiposity and muscle strength status who did not meet the full definition of dynapaenic abdominal obesity. Due to the limited sample size, further stratified analyses were not feasible. Therefore, subgroup findings should be considered exploratory and hypothesis‐generating.

## 5. Conclusion

We found a high prevalence of dynapaenic abdominal obesity in women diagnosed with breast cancer admitted for cancer surgery. Dynapaenic abdominal obesity was independently associated with poorer QoL, even after adjustment for age, income, education and physical activity. The assessment of muscle strength and body fat distribution, using simple and low‐cost measures, may help identify women with greater vulnerability to poorer QoL. Our findings add to the body of evidence which advocates early proactive interventions targeting dynapaenic obesity in breast cancer management protocols. Further longitudinal studies are needed to clarify the temporal relationships between dynapaenic abdominal obesity and QoL outcomes in women with breast cancer.

## Author Contributions

Conceptualisation: Taise Andrade da Anunciação and Lucivalda Pereira Magalhães de Oliveira.

Methodology: Taise Andrade da Anunciação and Lucivalda Pereira Magalhães de Oliveira.

Data curation: Taise Andrade da Anunciação, Lucivalda Pereira Magalhães de Oliveira and Maria Lúcia Varjão da Costa.

Formal analysis: Taise Andrade da Anunciação, Lucivalda Pereira Magalhães de Oliveira, Anna Karla Carneiro Roriz and Priscila Ribas Farias Costa.

Resources: Rosângela Passos de Jesus and Lucivalda Pereira Magalhães de Oliveira.

Writing–original draft: Taise Andrade da Anunciação, Lucivalda Pereira Magalhães de Oliveira, Priscila Ribas Farias Costa, Rosângela Passos de Jesus and Allain A. Bueno; writing–review and editing: Taise Andrade da Anunciação, Anna Karla Carneiro Roriz, Maria Lúcia Varjão da Costa, Viviane Sahade Souza, Rosângela Passos de Jesus, Priscila Ribas Farias Costa, Allain A. Bueno and Lucivalda Pereira Magalhães de Oliveira.

Visualisation: Rosângela Passos de Jesus, Priscila Ribas Farias Costa, Allain A. Bueno and Lucivalda Pereira Magalhães de Oliveira.

Supervision: Lucivalda Pereira Magalhães de Oliveira.

Project administration: Lucivalda Pereira Magalhães de Oliveira.

Funding acquisition: Rosângela Passos de Jesus, Priscila Ribas Farias Costa and Lucivalda Pereira Magalhães de Oliveira.

## Funding

The authors would like to thank the Coordination for the Improvement of Higher Education Personnel—Brazil (CAPES–Finance Code 001), which supports the Postgraduate Program in Food, Nutrition and Health of the School of Nutrition at the Federal University of Bahia.

## Ethics Statement

This study was conducted in accordance with the Declaration of Helsinki 1964 and with the ethical principles established by the Resolution 466 of 08 December 2012 of the National Health Council of Brazil. Ethical approval for this study was granted by the Ethics and Research Committee of the Bahian League Against Cancer Aristides Maltez Hospital—Protocol 2.950.264/2018. All participants voluntarily participated in this study and provided written informed consent.

## Consent

All authors gave their consent for this study to be published.

## Conflicts of Interest

The authors declare no conflicts of interest.

## Supporting Information

Additional supporting information can be found online in the Supporting Information section.

## Supporting information


**Supporting Information** Mean scores for the subdomains (physical, social/family, emotional, functional and the Breast Cancer Subscale) comprising the QoL questionnaire according to the descriptive characteristics of the women admitted for breast cancer surgery included in the study (Supporting Table 1).

## Data Availability

The data that support the findings of this study are available from the corresponding author upon reasonable request.
