# Supplementary material for: Dynapaenic Abdominal Obesity Is Associated With a Poorer Quality of Life in Women Diagnosed With Breast Cancer Awaiting Surgery
Source: J Obes. 2026 Oct 17;2026:4826660. doi: 10.1155/jobe/4826660 (PMC13676304; doi:10.1155/jobe/4826660)
Supplement: Supplementary file 1 [file JOBE-2026-4826660-s001.docx]

**Supplementary table 1:** subdomain mean scores of QoL questionnaires according to descriptive characteristics in women admitted to hospital for breast cancer surgery included in the investigation; Salvador, Bahia-Brazil.

| **Variables** | **FACT-B Domains** | | | | |
| --- | --- | --- | --- | --- | --- |
|  | **Physical** | **Social/**  **family** | **Emotional** | **Functional** | **Breast Cancer Subscale** |
|  | Mean (SD) | Mean (SD) | Mean (SD) | Mean (SD) | Mean (SD) |
| **Education** |  |  |  |  |  |
| High School or university | 22.5 (4.9) | 22.5 (3.8) | 19.8 (4.6) | 21.0 (5.3) | 25.8 (5.6) |
| Primary or secondary | 22.9 (4.4) | 20.5 (3.7) | 18.9 (4.5) | 19.2 (4.5) | 26.1 (5.6) |
| p-value | 0.71 | **0.01** | 0.33 | 0.06 | 0.74 |
| **Self-declared ethnicity** |  |  |  |  |  |
| non-BAME | 20.3 (5.4) | 19.9 (3.4) | 19.0 (4.5) | 16.0 (6.3) | 24.9 (6.3) |
| BAME | 23.1 (4.4) | 21.6 (3.8) | 19.3 (4.5) | 20.7 (4.4) | 26.1 (5.6) |
| p-value | **0.04** | 0.13 | 0.83 | **<0.01** | 0.44 |
| **Family income** |  |  |  |  |  |
| ≥ 2SM | 22.9 (4.7) | 22.9 (3.9) | 19.2 (4.1) | 20.1 (6.1) | 24.8 (5.4) |
| < 2 SM | 22.6 (4.7) | 21.2 (3.5) | 19.1 (4.8) | 20.0 (4.2) | 26.0 (5.7) |
| p-value | 0.81 | **0.03** | 0.95 | 0.90 | 0.35 |
| **Regular physical exercise** |  |  |  |  |  |
| Yes | 23.2 (4.3) | 21.0 (3.6) | 20.6 (4.4) | 20.1 (4.4) | 27.7 (4.5) |
| No | 22.6 (4.7) | 21.5 (3.9) | 18.8 (4.5) | 20.0 (5.1) | 25.4 (5.8) |
| p-value | 0.61 | 0.61 | 0.11 | 0.93 | 0.08 |
| **PG-SGA** |  |  |  |  |  |
| No risk | 23.4 (4.3) | 21.1 (3.8) | 19.5 (4.4) | 20.2 (4.7) | 26.2 (5.6) |
| At risk | 18.9 (4.7) | 22.8 (3.8) | 18.2 (5.1) | 18.9 (6.3) | 24.6 (5.2) |
| p-value | **<0.01** | 0.12 | 0.33 | 0.36 | 0.29 |
| **BMI** |  |  |  |  |  |
| Obesity | 22.4 (4.6) | 21.1 (3.6) | 18.2 (4.8) | 19.5 (4.9) | 24.6 (6.1) |
| Overweight | 23.1 (4.7) | 21.8 (4.1) | 20.6 (3.8) | 20.7 (4.9) | 27.8 (4.2) |
| p-value | 0.45 | 0.42 | **0.01** | 0.24 | **<0.01** |
|  |  |  |  |  |  |

Student's t-test. BAME: Black, Asian, and Minority Ethnic groups; SM: Minimum wage in Brazilian currency, equivalent to US$183.2 at the time of the study; PG-SGA: Patient-Generated Subjective Global Assessment; BMI: Body Mass Index. N=97
